# Cancer-Associated Venous Thromboembolic Disease: Anatomoclinical Aspects and Risk Factors

**DOI:** 10.7759/cureus.91276

**Published:** 2025-08-30

**Authors:** Imen Ketata, Aloulou S Samir, Abir Bouassida, Faten Dhouib, Manel Bahri, Hadil Chaari, Hamdani Moez, Rahma Kallel, Hayfa Rajhi

**Affiliations:** 1 Department of Medical Oncology, Faculty of Medicine, University of Sfax, Gabes University Hospital, Gabes, TUN; 2 Department of Cardiology, Faculty of Medicine, University of Sfax, Gabes University Hospital, Gabes, TUN; 3 Department of Radiotherapy, Faculty of Medicine, University of Sfax, Gabes University Hospital, Gabes, TUN; 4 Anatomopathology Laboratory, Faculty of Medicine, University of Sfax, Sadok Mkaddem Hospital, Djerba, TUN; 5 Medical Analysis Laboratory, Faculty of Medicine, University of Sfax, Gabes University Hospital, Gabès, TUN

**Keywords:** cancer, oncology, risk factor, thrombosis, venous thromboembolic disease

## Abstract

Background

Cancer represents an independent risk factor for venous thromboembolic disease (VTE). The main objective of this study is to analyze the cancer profile as well as the related VTE risk factors.

Materials and methods

A retrospective, descriptive, and analytical study was conducted in the Gabes University Hospital from January 2017 to May 2021. The data study collection focused on patients hospitalized with VTE-associated cancer. Input data and statistical analysis were performed using IBM SPSS Statistics for Windows, Version 26 (Released 2019; IBM Corp., Armonk, New York, United States).

Results

The study involved 135 patients. The average age of the patients was 59.21 years, ranging from 17 to 94 years, and the sex ratio of 1.81. Lung, pancreatic, and bladder cancers significantly increased the risk of VTE by 1.48 (95% CI [1.19-1.85]), 2.60 (95% CI [1.31-5.3]), and 2.61 (95% CI [1.26-5.4]), respectively. Among the patients with metastatic cancer, the risk of developing VTE was 1.55 times higher than patients without metastatic cancer (95% CI [1.1-2.1]). Small cell, squamous cell, and urothelial carcinoma significantly increased (p<0.001) the risk of VTE occurrence by 2.79 (95% CI [1.69-4.6]), 2.41 (95% CI [1.38-4.13]), and 3.03 (95% CI [1.33-6.8]), respectively.

Conclusion

Among the patients with metastatic cancer, the risk of developing VTE was higher than patients without metastatic cancer. In addition, small cell, squamous cell, and urothelial carcinoma significantly increased the risk of VTE. Problems and knowledge of the VTE risk factors among cancer patients allow for cancer management improvement as well as avoiding further thromboembolic complications.

## Introduction

The relationship history between cancer and venous thromboembolic disease (VTE) dates since 1823 by Bouillaud. Currently, the VTE is continuously increasing and represents a main clinical issue [1,2]. VTE is likely to complicate the evolution of cancer patients in 10 to 15% of cases. In addition, cancer can be the origin of VTE in 20% of cases. VTE has even potential to constitute the first sign of tumor pathology. Indeed, this association complicates the management of both diseases [3]. Obviously, the association of cancer and thrombosis is not fortuitous. Cancer remains an independent risk factor for thrombosis by interfering with Rudolf Virchow's triad. In fact, cancer is responsible for venous stasis, endothelial injury, and hypercoagulability. Moreover, partial or total compression of veins, immobility, sarcopenia secondary to repeated hospitalizations, and anticancer treatments lead to venous stasis [4]. Cancer cells directly invade the vascular wall on which they adhere and release cytokines such as TNF-α, thus promoting endothelial injury. In addition, central catheterization, chemotherapy (CT), and radiotherapy (RT) induce vascular injury and toxicity [4]. Furthermore, tumors cause hypercoagulability through poor-quality neoplastic angiogenesis, a pro-aggregating effect, and the reduction of natural anticoagulant proteins (Protein S and C). Therefore, cancer favors coagulation through factors that themselves facilitate tumor progression and invasion. The occurrence of VTE is a poor prognostic factor among cancer patients and represents the second cause of death among them [5]. The main objective of this study is to describe the anatomical characteristics of VTEs when affected by cancer and to identify the risk factors for the development of VTEs among cancer patients.

## Materials and methods

Study cohort

An analytical cohort study with retrospective data collection was conducted in the Gabes University Hospital between January 2017 and May 2021. The study focused on patients hospitalized with cancer.

Inclusion and exclusion criteria

The inclusion criteria for diagnosing cancer were based on: Histologically confirmed solid cancer or Hodgkin or non-Hodgkin lymphoma and being associated with VTE, whether or not it was revealing or not, synchronous or metachronous. However, patients were excluded from the study if they met any of the following criteria: Histologically unconfirmed cancer or a hematological malignancy other than non-Hodgkin lymphoma and Hodgkin disease, a suspicion of VTE not confirmed by imaging, or incomplete records.

Patients with cancer-associated VTE were selected from the different hospital departments such as pulmonology, internal medicine, cardiology, and medical oncology. Data on clinical, paraclinical, and therapeutic conditions of patients were retrieved from medical records. Clinical data collected included demographics (age, sex, medical and surgical history), comorbidities, SARS-CoV-2 infection, cancer characteristics (disease location, histological type, cancer stage, histological grade, stage, and systemic treatment of cancer), and the anatomoclinical characteristics of VTE.

Statistical analysis

Data entry and statistical analysis were performed using IBM SPSS Statistics for Windows, Version 26 (Released 2019; IBM Corp., Armonk, New York, United States). Qualitative data were expressed by the observed numbers and in relative proportions (percentage). For quantitative variables, the study of data distribution was conducted by normality tests. They were expressed as mean and confidence interval. In order to determine the anatomical, histological, and therapeutic cancer risk factors for the development of VTE, the relative risk (RR) and the confidence interval (95% CI) were analyzed to assess the strength of the associations studied using the chi-square test.

## Results

During the study period, 1600 patients were examined with cancer disease in the different hospital departments. Only 135 patients developed VTE. Thus, the VTE prevalence among cancer patients was 8.43% in our population.

Patient demographic characteristics

**Figure 1:**
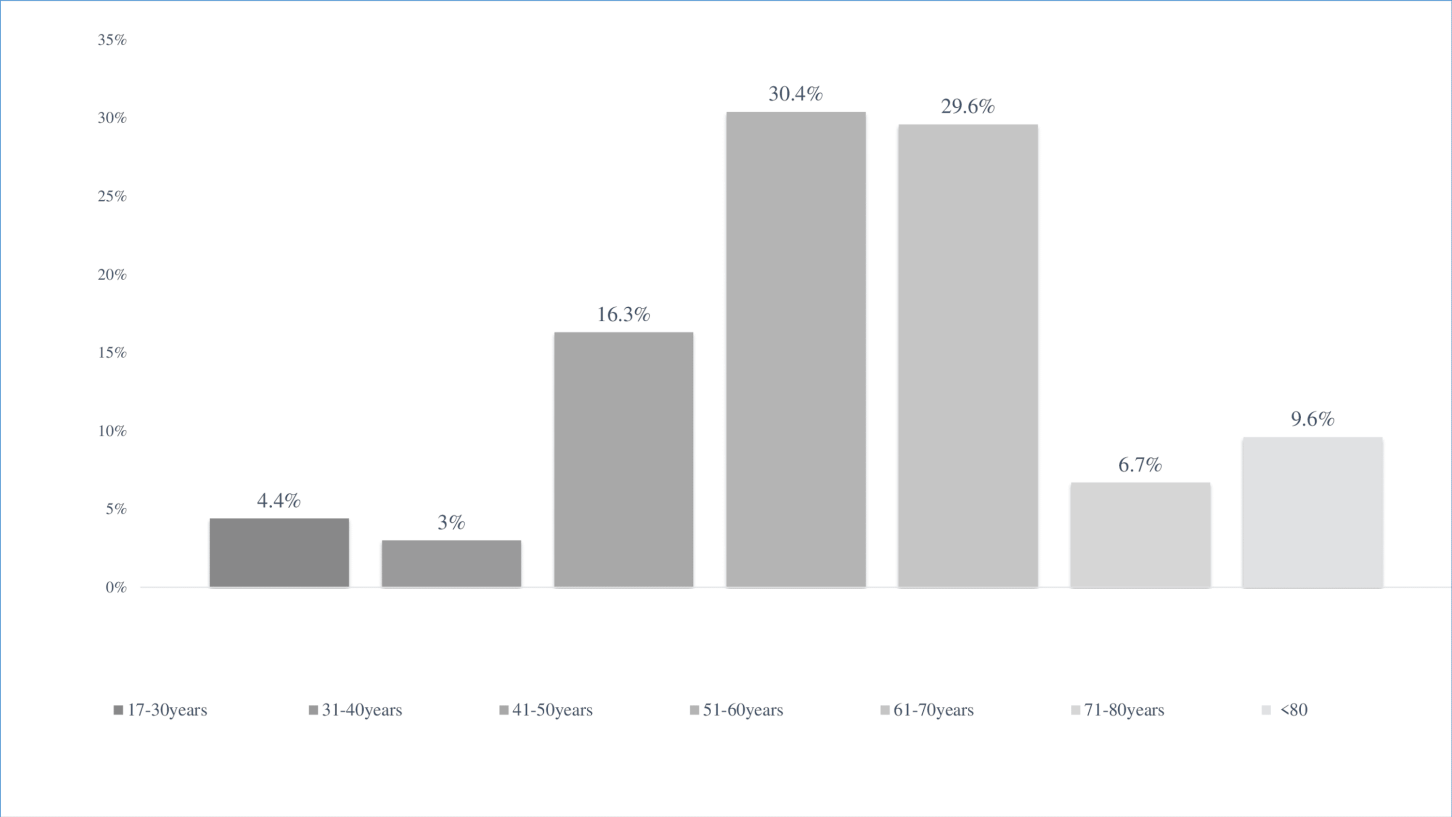
Distribution of patients according to age

Figure 1 shows the patient cancer VTE distribution according to age. The mean age of patients (n=135) was 59.21 years, ranging from 17 to 94 years. A peak incidence (60%) of the VTE and cancer association was observed in the age range between 50 and 70 years. A male predominance was noted with a sex ratio equal to 1.81. Eight patients (5.92%) had a documented personal history of VTE diagnosed and treated before the diagnosis of the primary cancer. The mean delay was 69.75 months (6-420 months) or 5 years and 8 months. Half of these patients developed deep vein thrombosis (DVT) of the lower limbs (50 %), 37.50% pulmonary embolism (PE) and 12.5% renal vein thrombosis. Regarding classic VTE risk factors, 20% of patients were obese and 0.74% had thrombophilia. In the patients' cohort, 18 had a history of pneumonia due to SARS-CoV-2 infection, i.e., 13.33%.

Tumor characteristics

Table 1 summarizes the different characteristics of cancers in patients with VTE (n=135). The three most common cancer locations were: pulmonary, pancreatic and breast, with 54 (40%), 9 (6.66%) and 9 (6.66%), respectively. Five cases of pulmonary locations had Pancoast-Tobias syndrome. Most cancers were metastatic with 91 (67.4%), followed by localized and locally advanced cancers, representing 23 (17%) of cases and 21 (15.6%) of cases, respectively. Stage IV was the most noted representing 67.4%. Adenocarcinoma was the most observed histological type with 55 (40.74%). Cancers were in the majority of cases moderately differentiated in 84 (62.22%) cases. Undifferentiated and well-differentiated cancers represented 38 (28.14%) and 13(9.62%) of cases, respectively. Treatment was based on CT 102 (75.55%) of cases. The most commonly used CT protocols were platinum salt-Gemcitabine in 28 cases (27.45%), platinum salt-5 Fluorouracil in 14 cases (13.72%) and platinum salt-Etoposide in 13 cases (12.74%). Therapeutic abstention was decided in 12 patients due to a profound deterioration in their general condition, with the use of palliative care aimed at relieving physical pain and other symptoms.

**Table 1:** Tumor characteristics among patients with thromboembolic disease (n=135)

Location	N	%
Lung	54	40
Breast	9	6.66
Pancreas	9	6.66
Kidney	8	5.92
Bladder	6	4.44
Cavum	6	4.44
Colon	5	3.7
Liver	4	2.96
Uterus	4	2.96
Ovary	4	2.96
Large cell lymphoma	4	2.96
Stomach	3	2.22
Gallbladder	3	2.22
Bone tumor	3	2.22
Parotid	2	1.48
Larynx	2	1.48
Thyroid	2	1.48
Hodgkin's lymphoma	2	1.48
Malignant glioma	2	1.48
Esophagus	1	0.74
Rectum	1	0.74
Prostate	1	0.74
Histological type		
Adenocarcinoma	55	40.74
Small cell carcinoma	17	12.59
Squamous cell carcinoma	14	10.37
Clear cell carcinoma	7	5.18
Urothelial carcinoma	12	8.88
Undifferentiated carcinoma of the nasopharynx	6	4.44
Infiltrating ductal carcinoma	6	4.44
Ductal carcinoma in situ	4	2.96
Cancer grade		
Well differentiated	13	9.62
Moderately differentiated	84	62.22
Undifferentiated	38	28.14
Cancer treatment		
Chemotherapy	102	75.55
Surgery	35	25.92
Radiotherapy	25	18.51
Targeted therapy	9	6.66
Concomitant chemo-radiotherapy	6	4.44
Hormone therapy	6	4.44
	N	%
Location		
Lung	54	40
Breast	9	6.66
Pancreas	9	6.66
Kidney	8	5.92
Bladder	6	4.44
Cavum	6	4.44
Colon	5	3.7
Liver	4	2.96
Uterus	4	2.96
Ovary	4	2.96
Large cell lymphoma	4	2.96
Stomach	3	2.22
Gallbladder	3	2.22
Bone tumor	3	2.22
Parotid	2	1.48
Larynx	2	1.48
Thyroid	2	1.48
Hodgkin's lymphoma	2	1.48
Malignant glioma	2	1.48
Esophagus	1	0.74
Rectum	1	0.74
Prostate	1	0.74
Histological type		
Adenocarcinoma	55	40.74
Small cell carcinoma	17	12.59
Squamous cell carcinoma	14	10.37
Clear cell carcinoma	7	5.18
Urothelial carcinoma	12	8.88
Undifferentiated carcinoma of the nasopharynx	6	4.44
Infiltrating ductal carcinoma	6	4.44
Ductal carcinoma in situ	4	2.96
Cancer grade		
Well differentiated	13	9.62
Moderately differentiated	84	62.22
Undifferentiated	38	28.14
Cancer treatment		
Chemotherapy	102	75.55
Surgery	35	25.92
Radiotherapy	25	18.51
Targeted therapy	9	6.66
Concomitant chemo-radiotherapy	6	4.44
Hormone therapy	6	4.44
Location		
Lung	54	40
Breast	9	6.66
Pancreas	9	6.66
Kidney	8	5.92
Bladder	6	4.44
Cavum	6	4.44
Colon	5	3.7
Liver	4	2.96
Uterus	4	2.96
Ovary	4	2.96
Large cell lymphoma	4	2.96
Stomach	3	2.22
Gallbladder	3	2.22
Bone tumor	3	2.22
Parotid	2	1.48
Larynx	2	1.48
Thyroid	2	1.48
Hodgkin's lymphoma	2	1.48
Malignant glioma	2	1.48
Esophagus	1	0.74
Rectum	1	0.74
Prostate	1	0.74
Histological type		
Adenocarcinoma	55	40.74
Small cell carcinoma	17	12.59
Squamous cell carcinoma	14	10.37
Clear cell carcinoma	7	5.18
Urothelial carcinoma	12	8.88
Undifferentiated carcinoma of the nasopharynx	6	4.44
Infiltrating ductal carcinoma	6	4.44
Ductal carcinoma in situ	4	2.96
Cancer grade		
Well differentiated	13	9.62
Moderately differentiated	84	62.22
Undifferentiated	38	28.14
Cancer treatment		
Chemotherapy	102	75.55
Surgery	35	25.92
Radiotherapy	25	18.51
Targeted therapy	9	6.66
Concomitant chemo-radiotherapy	6	4.44
Hormone therapy	6	4.44

Characteristics of VTE

The development of VTE was symptomatically observed in 102 (75.55%) of cases, which was revealed and diagnosed during the progression of the cancer in 32 (23.75%) and 70 (51.80%) of cases, respectively. Furthermore, VTE was discovered incidentally in 33 (24.44%) of cases, which was concomitant with the discovery of the cancer, was during the extension assessment, and was diagnosed incidentally during a follow-up consultation in seven (5.18%), nine (6.67%), and 17 (12.59%) of cases, respectively. In the patients' cohort, the majority had a metachronous VTE (98, 72.59%) compared to the cancer. Concerning the four patients with a VTE before the cancer, the average delay was 15 days to one month (1 - 2 months). About patients with a metachronous VTE, the average delay between the diagnosis of cancer and the occurrence of VTE was 13.43 months. The mean time between VTE and surgery, VTE and RT, and VTE and CT was 18.97 months (1-180 months), 9.8 months (10 days - 72 months), and six months and 21 days (1 - 90 days), respectively. In the majority of cases, VTE occurred within the first three months (65.2%). All patients who had concomitant CT-RT developed VTE after this treatment with a mean time of one month. Among the nine patients who received targeted therapy, two of them had VTE occurring before the administration of this treatment. Furthermore, seven patients had VTE after. The mean time between targeted therapy and VTE was three months (15 days - 9 months). For hormone therapy (HT) (n=6), only one patient had VTE before HT. The other ones (five patients) had VTE after the start of HT. The average time was 35.6 months (3 months - 100 months).

**Figure 2:**
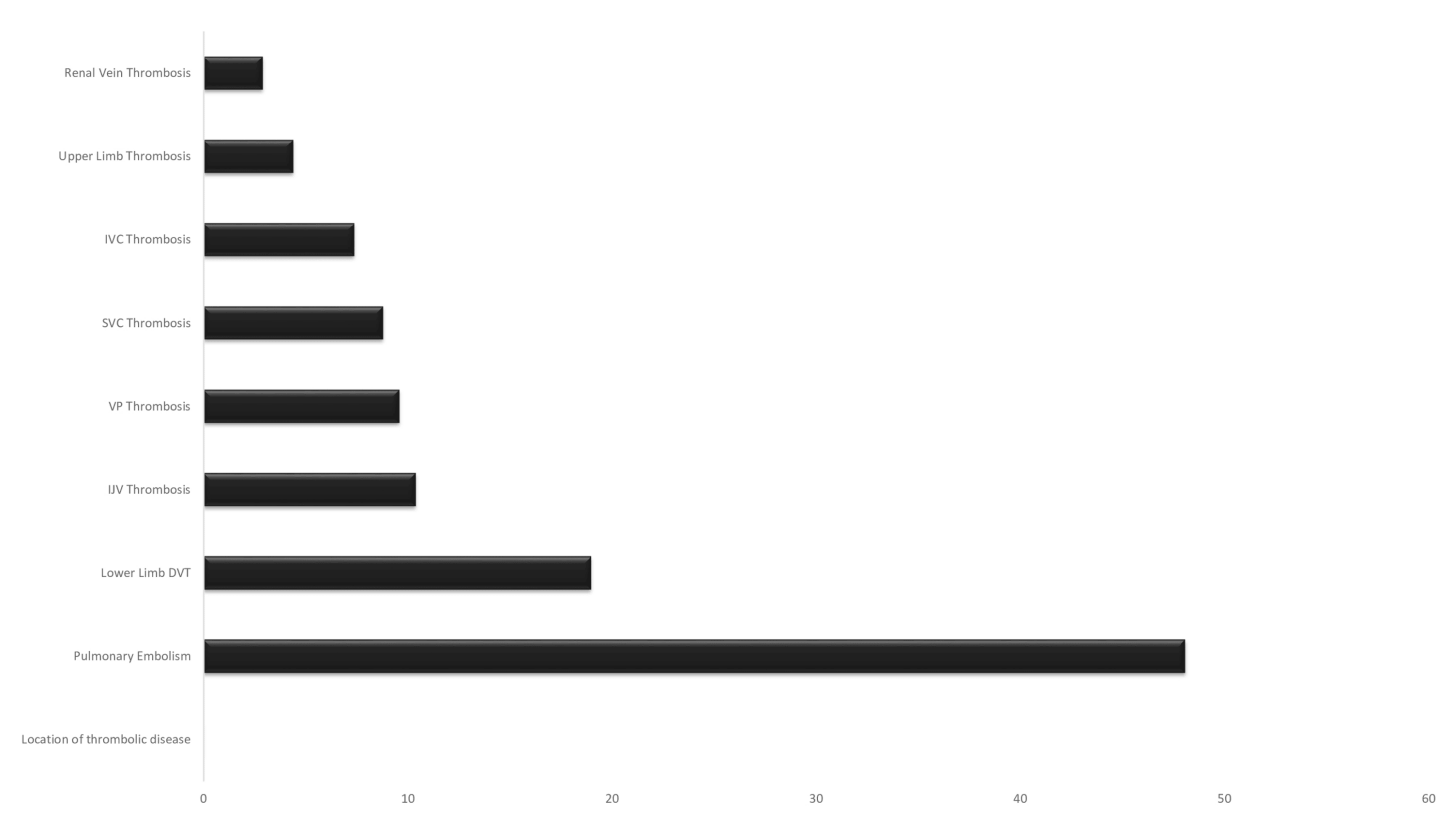
Location of thrombolic disease IJV: Internal jugular vein, VP: portal vein, SVC: superior vena cava, IVC: inferior vena cava

PE was the most common in the study series, with 48.14% of cases, followed by DVT of the lower limbs with 26 cases (19.25%) and then internal jugular vein thrombosis (IJV) with 14 patients (10.37%), as shown in Figure 2.

Table 2 illustrates VTE-affected sites. VTE associations were observed in 11.10% of cases. PE was often segmental and subsegmental in 41 (30.30%) of cases. Doppler ultrasound of the lower limbs demonstrated unilateral and bilateral DVT in 125 (92.30%) and 10 (7.70%) of cases, respectively. DVT was proximal in the majority of cases (130, 96.17%). Furthermore, it was distal in five (3.83%) of cases.

**Table 2:** VTE with more than one affected site (n=15) PE: Pulmonary embolism, DVT: Deep vein thrombosis, SVC: Superior vena cava, IJV: Internal jugular vein, IVC: Inferior vena cava

Location	N	%
PE and DVT of the lower limbs	4	2.96
PE and SVC thrombosis	3	2.22
SVC thrombosis and upper limb thrombosis	2	1.48
IJV thrombosis and cerebral thrombophlebitis	2	1.48
Thrombosis of the IJV, IVC and cerebral thrombophlebitis	2	1.48
Thrombosis of the IJV and subclavian vein	1	0.74
Thrombosis of the SVC, IJV and subclavian vein	1	0.74

The most common location was popliteal vein thrombosis in seven cases. As for IJV thrombosis, it was present in two cases, associated with the superior vena cava (SVC) thrombosis in two cases, associated with cerebral thrombophlebitis in two cases and bilateral IJV thrombosis was observed in one case. SVC thrombosis extended to the subclavian and internal jugular veins was noted in only one patient. Four patients had very tight floating inferior vena cava (IVC) thrombosis. Respecting the renal vein, the thrombosis was on the right in all cases. Thoracic-abdominal-pelvic CT identified PV thrombosis with the presence of portal cavernoma in two cases. The anatomical characteristics of VTE in cancer patients are detailed in Table 3.

**Table 3:** Anatomical characteristics of thrombosis among cancer patients (n=135)

Site of thrombosis			N	%
Pulmonary embolism (n=66)		Truncular	3	4.54
		Lobular	11	16.66
		Segmental	9	13.63
		Subsegmental	13	19.69
		Segmental and subsegmental	20	30.30
		Lobar and segmental	4	6.06
		Lobar + segmental and subsegmental	3	4.54
		Troncular + lobar + segmental and subsegmental	2	3.03
Lower limb DVT (n=18)		Popliteal vein thrombosis	7	26.92
		Femoral vein thrombosis	3	11.53
		Thrombosis of the iliofemoral axis	3	11.53
		Thrombosis of the femoropopliteal axis	3	11.53
		Thrombosis of the iliofemoral-popliteal axis	1	3.84
		Anterior tibial vein thrombosis	1	3.84
Upper limb DVT (n=6)	Thrombosis of the axillary vein and subclavian vein extending to the brachiocephalic venous trunk		3	50.00
	Thrombosis of the subclavian vein, axillary vein, basilic vein and brachial vein		3	50.00

The analytical study showed that lung, pancreatic and bladder cancer significantly increased the risk of VTE by 1.48 (95% CI [1.19-1.85]), 2.60 (95% CI [1.31-5.3]) and 2.61 (95% CI [1.26-5.4]), respectively (Table 4). Only metastatic cancer and stage IV significantly increased the risk of VTE development by 1.55 (95% CI [1.1-2.1]) and 1.19 (95% CI [1.04-1.35]). Regarding the histological type, small cell carcinoma, squamous cell carcinoma and urothelial carcinoma significantly increased the risk of VTE in cancer patients by 2.79 (95% CI [1.69-4.6]), 2.41 (95% CI [1.38-4.13] and 3.03 (95% CI [1.33-6.8]), respectively, as shown in Table 5.

**Table 4:** The relative risk of thromboembolic disease

	RR	IC
Location of cancer		
Lung	1.48	1.19-1.85
Pancreas	2.60	1.31-5.3
Breast	0.28	0.15-0.58
Kidney	1.83	0.95-3.55
Bladder	2.61	1.26-5.4
Cavum	1.71	0.8-3.6
Colon	0.32	0.13-1.1
Liver	0.98	0.36-2.55
Uterus	2.10	0.88-5.39
Stage of cancer dissemination		
Local	1.18	0.77-1.92
Locally advanced	0.48	0.31-0.75
Metastatic	1.55	1.1-2.1
Cancer stage		
Stage I	1.91	0.81-4.48
Stage II	1.81	0.51-1.27
Stage III	0.62	0.42-0.9
Stage IV	1.19	1.04-1.35

**Table 5:** Histological type, cancer grade and treatment

Histological type	RR	IC
Adenocarcinoma	0.68	0.49-0.95
Small cell carcinoma	2.79	1.69-4.6
Squamous cell carcinoma	2.41	1.38-4.13
Clear cell carcinoma	1.89	0.86-4.15
Urothelial carcinoma	3.03	1.33-6.8
Undifferentiated carcinoma of the nasopharynx	1.50	0.6-3
Infiltrating ductal carcinoma	0.24	0.11-0.54
Ductal carcinoma in situ	0.33	0.1-1.94
Cancer grade	RR	IC
Differentiated	0.76	0.44-1.3
Moderately differentiated	0.95	0.8-1.09
Undifferentiated	1.27	0.94-1.71
Treatment	RR	IC
Surgery	0.92	0.69-1.23
Radiotherapy	0.96	0.63-1.4
Chemotherapy	1.09	0.98-1.21
Concomitant chemo-radiotherapy	0.49	0.2-1.19
Targeted therapy	1.56	0.9-2.7

## Discussion

In this study, we evaluated the relative risk of VTE occurrence in cancer patients by studying the anatomical, histological, and therapeutic characteristics of cancer. VTE remains a common and serious complication in cancer patients. In this current study, the prevalence of VTE was 8.4%. Several bibliographic data support the importance of VTE prevalence in cancer patients than in non-cancer patients [6]. It was ambivalent between 18.2% and 57.1% in neoplastic diseases and between 4.2% and 37.8% in the absence of neoplasia [6]. The variance in VTE risk in cancer patients is attributed to the anatomical and histological characteristics of cancer and its therapeutic protocol. In these cohorts’ series, lung, pancreatic, and bladder cancer were significantly associated with the risk of VTE. Similar to the current study, different series estimated that lung cancer was the highest contributor to VTE [7]. Moreover, several publications showed that pancreatic cancer had a higher risk of VTE occurrence [8]. Lung cancer represented an etiology of VTE in 7.5% and 21% of cases [5]. Furthermore, pancreatic cancer was a cause of VTE in 4% to 27.1% of cases [9]. The prevalence of breast and kidney cancer was also high, ranging from 9.1% to 13.9%, and 1.5% to 24% respectively. However, those of bladder and brain cancer remained lower, between 3.4% and 4%, and 1.7% and 3.5%, respectively [9]. In the case of lung cancer, there were genetic mutations (K-ras) contributing to VTE. Moreover, those who generally have a slower-growing and more indolent malignancy, such as breast cancer and prostate cancer, had much lower incidences of developing VTE (1%) [10,11]. It is shown that some pretumoral cells, under the influence of coagulation factors, will promote tumor migration and invasion and consequently an increased risk of tumor thrombus [12]. Distant metastases of cancer at diagnosis have been associated with a higher risk of VTE compared to localized disease [13]. As an aligned, we observed that metastatic stage and stage IV significantly increase the risk of VTE development by 1.55 (95% CI [1.1-2.1]) and 1.19 (95% CI [1.04-1.35]), respectively. This observation was shared by several publications that confirmed that stages III and IV were significantly associated with an increased risk of VTE occurrence [14-16]. A locally advanced stage has also been documented as a risk of VTE development during cancer (HR 2.6, 95% CI, 1.7-4.0) [16]. These results expound the mobility reduction and major comorbidities in patients with metastases and the increased use of different antitumor treatments. Adenocarcinoma was the most observed histological type in this study (40.7%). This result complies with the literature data. It was observed in 77% of cases in the study conducted by Alcalay et al. [17]. ADKs appear to be particularly the most likely to cause VTE and were twice as emboligenic as other histological types [18]. The risk of each histological type differs from one series to another, depending on the number of samples included and the location of the cancer. In contrast to the current study, non-small cell lung cancer had a risk of VTE twice as high as non-small cell carcinoma was observed in previous studies. In other series, the most likely histological type to cause VTE was clear cell carcinoma [19,20]. However, the histological types most observed with risk for VTE were small cell carcinoma, squamous cell carcinoma, and urothelial carcinoma. The cumulative probability of developing VTE was significantly higher among patients with high-grade tumors than in those with low-grade tumors [11,18]. Systemic cancer treatments also increase the risk of VTE occurrence by six to seven times, with an annual incidence of 11%. In contrast to this study, it is important to note that surgical treatment mainly allows the occurrence of VTE in cancer by the disruption of coagulation factors. It appears that CT has overtaken (twice) the VTE risk occurrence among cancer patients [19]. This risk is attributed to the cytotoxicity of CT that damages endothelial cells and increases the generation of thrombin, tissue factors, and von Willebrand factors. In addition, targeted therapy (including Bevacizumab) and HT (including Tamoxifen) have been correlated with a VTE risk occurrence. The National Surgical Adjuvant Breast and Bowel Project showed that the relative risk of VTE with tamoxifen was 2.5 to 3 times higher than placebo [20]. It is unanimously recommended to use anti-aromatase among people with VTE risk factor occurrence more than Tamoxifen, since it is less thrombogenic. It is now imperative to reconsider or revise the current cancer therapeutic strategies to avoid the occurrence of VTE. However, several other studies have not shown a significant association between VTE and RT, despite it inducing inflammation, endothelial activation, cell death, and accelerating the coagulation cascade and platelet thrombus formation.

As a rule, VTE can be examined incidentally among cancer patients during a follow-up assessment or in the presence of clinical symptoms. Mostly, VTE occurs metachronously to the cancer. Moreover, it can also be synchronous or before it (or revealing) [21,22]. Chang et al. observed a significant superiority of asymptomatic forms of PE among cancer patients compared to the general population (54.5% vs. 13.2%) (p (p<0.001) [23]. Hence, an immediately bilateral, idiopathic, or recurrent VTE should lead to a systematic search for cancer. VTE develops typically during the first year following cancer diagnosis [24]. In this study, most VTEs occurred particularly during the first three months of cancer diagnosis. This peak of VTEs during the first three months is probably related to a high risk of VTE occurrence during anticancer treatment. The most frequent VTEs in the current study were PE (48.14%), lower limb DVT (19.25%), and IJV thrombosis (10.4%). The sites of VTE are ambivalent depending on the cancer locations. Distal, proximal, and mixed locations of PE were also heterogeneous in the literature. The incidence of lower limb DVT seems to increase from 4 to 7 times among cancer patients [25]. In the literature, unilateral DVT was dominant in cases of associated cancer [25]. Bilateral DVT was noted in 8.5% and 7.14% of cases in a French and Moroccan study, respectively [25]. Proximal axes are more frequently affected than distal axes among cancer patients in several publications [26]. However, thrombosis of the IJV and renal vein was rare in several series and may be part of a paraneoplastic syndrome. Involvement of the axillary, subclavian, and brachial veins was most marked during neoplasia. The VTE occurrence during cancer is characterized by the recurrence of thrombosis episodes and increased resistance to usual treatment, which worsens the prognosis and increases the mortality rate.

This study had several limitations: a retrospective study, a small sample size, and a single-center study with a population representative only of the southern Tunisia governorates region. This population was characterized by the predominance of breast and lung cancers. The latter cancer was particularly frequent, revealed in a context of tobacco scourge as well as air pollution closely linked to local chemical industries. In addition, this population was stamped by the frequency of advanced metastatic cancers, which were more affected by thromboembolic disease.

## Conclusions

The current study analyzed the cancer profile as well as the related VTE risk factors. Among the patients with metastatic cancer, the risk of developing VTE was higher than patients without metastatic cancer. In addition, small cell, squamous cell, and urothelial carcinoma significantly increased the risk of VTE. It is to be noted that the relationship between cancer and VTE is well-known and documented. This association is morbid and risks making cancer management more complex.

Therefore, the management of VTE associated with cancer must be multidisciplinary, involving a medical oncologist, radiotherapist, cardiologist, angiologist, and vascular surgeon. Direct oral anticoagulants are a recent innovation in medical treatment and are now widely prescribed. Moreover, larger series are needed to identify risk factors for VTE developing related to cancer characteristics, prevention strategies and basically to assess the anatomoclinical specificities of VTE among cancer patients.
